# Fate of So‐Called Biodegradable Polymers in Seawater and Freshwater

**DOI:** 10.1002/gch2.201700048

**Published:** 2017-06-23

**Authors:** Amir Reza Bagheri, Christian Laforsch, Andreas Greiner, Seema Agarwal

**Affiliations:** ^1^ Macromolecular Chemistry II and Bayreuth Centre for Colloid and Interfaces University of Bayreuth Universitätsstraße 30 95440 Bayreuth Germany; ^2^ Department of Animal Ecology I and BayCEER University of Bayreuth Universitätsstraße 30 95440 Bayreuth Germany

**Keywords:** biodegradable polyesters, freshwater, microplastics, seawater

## Abstract

The stability of polymers with C—C and stable C—heteroatom backbones against chemicals, hydrolysis, temperature, light, and microbes has challenged society with the problem of accumulation of plastic waste and its management worldwide. Given careless disposal of plastic waste, large amounts of plastic litter accumulate in the environment and disintegrate into microplastics. One of the questions frequently raised in the recent times is if so‐called biodegradable polymers can substitute conventional polymers for several applications and help to tackle this challenge. The answer is not so simple as biodegradability is a certified property occurring only under certain environmental conditions and therefore requires systematic study. As a first step, this study focusses on comparative degradation studies of six polymers (five taken from the so‐called biodegradable polyesters, including poly(lactic‐*co*‐glycolic acid) (PLGA), polycaprolactone (PCL), polylactic acid (PLA), poly(3‐hydroxybutyrate) (PHB), Ecoflex, and one well‐known non‐degradable polymer poly(ethylene terephthalate) (PET) in artificial seawater and freshwater under controlled conditions for 1 year. Only amorphous PLGA shows 100% degradation as determined by weight loss, change in molar mass with time, NMR, electron microscopy, and high‐performance liquid chromatography. This is a step forward in understanding the degradability of polyesters required for the design of environmentally friendly novel polymers for future use.

## 1 Introduction

The production of synthetic polymers and their everyday use has been increased dramatically in the last three decades.^1^, ^2^, ^3^ One of the important properties of polymers that make them interesting for engineering and commercial applications is the stability against chemicals, hydrolysis, temperature, light, microbes, etc. This essential characteristic of polymers challenges society in dealing with the problem of plastic waste entering the environment via careless disposal. The disintegration of plastic debris into microplastics^4^, ^5^ and the accumulation in the world's oceans and freshwater (FW) ecosystems have raised concerns since large plastic debris and microplastics may elicit adverse effects in biota. Stable polymers such as polyethylene, polypropylene, polystyrene, poly((meth)acrylates), aromatic polyesters, and polyamides with either strong C—C or C—heteroatom backbone are considered to be the main sources of environmental pollution with microplastics.^6^, ^7^, ^8^, ^9^, ^10^, ^11^, ^12^ In contrast, conventional biodegradable polymers (aliphatic and aliphatic–aromatic polyesters), such as polylactic acid (PLA), polycaprolactone (PCL), lactide–glycolide copolymers, have not attracted the main attention as sources of microplastics. Probable reasons could be the limited use of these polymers and the common misconception that they would degrade under any environmental conditions. In reality, however, biodegradable polymers degrade only under certain conditions (temperature, humidity, light, oxygen availability, and microorganisms).^13^, ^14^, ^15^, ^16^, ^17^, ^18^, ^19^, ^20^, ^21^ Therefore, just because they are termed as “biodegradable polymers” does not rule out their potential contribution to environmental contamination. Only a limited number of studies focus on the degradability of biodegradable polymers in water sources and show disputable results as different methods were used for following the degradability.^22^, ^23^ In many studies, samples were placed in a perforated basket in seawater (SW), and weight change of the leftover material is noted making no demarcation between the weight loss due to biodegradation or simply due to the disintegration of the samples to microplastics getting lost in the sea as secondary microplastics.^24^, ^25^, ^26^, ^27^, ^28^, ^29^ The studies under controlled conditions using standard characterization techniques are required to understand the degradation behavior and to provide a comparative basis. Tsuji et al. carried out comparative degradation of PCL, PLA (amorphous and crystalline) and poly(3‐hydroxybutyrate) (PHB) at 25 °C for predetermined periods in seawater from the Pacific Ocean.^30^ PCL was 25% degraded in 10 weeks whereas PHB only by 9%.

In this work, we investigated the degradation behavior of five polymers from the class of biodegradable polymers (poly(lactic‐*co*‐glycolic acid) (PLGA), PCL, PLA, PHB, and Ecoflex), and a well‐known non‐degradable commercially useful polymer poly(ethylene terephthalate) (PET) for comparison purpose) under controlled conditions in the laboratory in artificial SW and FW (Figures S1–S3 and Tables S1 and S2, Supporting Information). Interestingly, 100% degradation was observed only for PLGA, whereas PCL, PLA, and PET did not degrade at all.

## 2 Results

**Figure 1:**
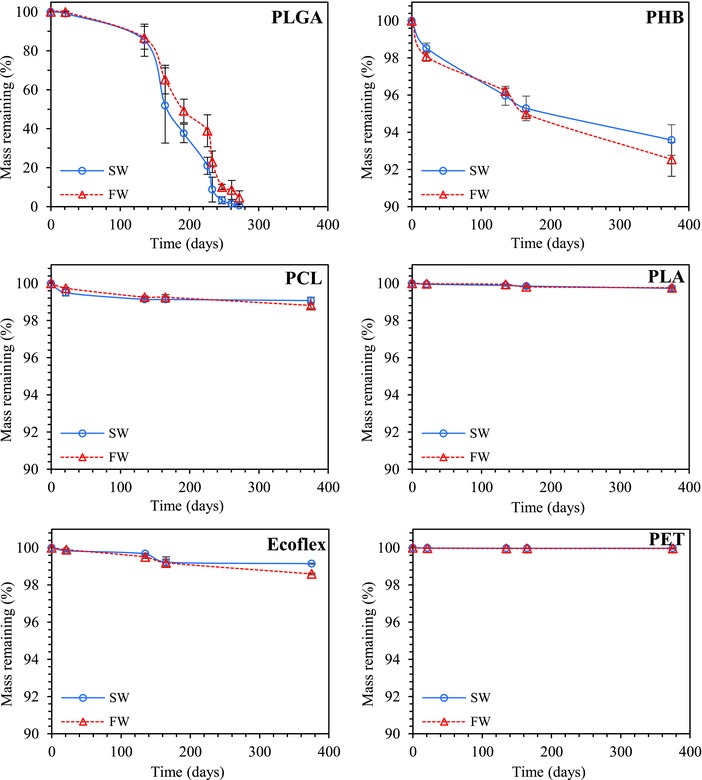
Mass losses of films made of PLGA, PCL, Ecoflex, PLA, PHB, and PET incubated in a thermostatic chamber at 25 °C in artificial seawater (SW) and freshwater (FW).

Films of PLGA, PCL, PHB, PLA, Ecoflex, and PET with an average thickness of 320 ± 20 μm (1.2 cm × 1.2 cm) were fabricated by hot pressing the corresponding granulates. The degradation studies were carried out in SW and FW under controlled conditions in a thermostatic chamber at 25 °C and under fluorescence light (16 h light and 8 h dark) for 1 year. The water was refreshed every 2 weeks. Mass loss of polymers at different time intervals has been presented in **Figure** **1**. Interestingly, PLGA showed the highest rate of degradation and completely degraded in ≈270 d. The degradation of PLGA took place in two steps (Figure 1). In the beginning, till about 135 d, a slow degradation (0.11 wt% per day) was observed. After this, the degradation rate was increased to ≈0.62 wt% per day. PHB degraded ≈8.5% in 1 year. The rate of degradation was about 0.027 wt% per day till 135 d and decreased to 0.01 wt% per day afterward. All other so‐called (bio)degradable polymers (PCL and PLA) did not show any significant degradability under tested conditions. Degradation rates of polymers were relatively same in FW and SW (Figure 1).

**Figure 2:**
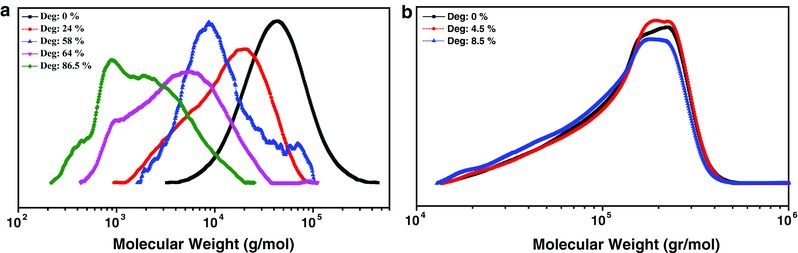
Characterization of films before and after degradation. GPC chromatograms of a) PLGA b) PHB.

PLGA degradation was followed in detail as it was the only polymer showing significant degradation. The molar mass of the polymer was monitored by gel permeation chromatography (GPC) at different time intervals. The GPC measurements showed significant changes in molar mass of the PLGA over time. The original PLGA showed a unimodal peak in GPC (**Figure** **2**a). The FW and SW exposed PLGA showed bi‐ and multimodal peaks in GPC for partially degraded samples indicating the dispersity in macromolecular chain lengths obviously due to the backbone scission. Although it was not possible to quantify the molar masses by GPC once degradation started due to multimodal peaks, there was a clear shift of molar mass to the low‐molar‐mass region with bi‐ and multimodal peaks. This is a distinct indication of degradation. The decrease in molar mass also hinted for bulk degradation. The degradation at ester linkages all throughout the bulk of the material leads to the formation of low‐molar‐mass oligomers, showing molar mass shifts and appearance of multimodal peaks in GPC. PHB showed degradation by surface erosion as no change in molar mass in GPC after even 8.5% mass loss (Figure 2b) was observed. Comparison between GPC results of degraded polymers in SW and FW did not demonstrate any meaningful differences. In general, polyester degradation takes place in two steps: scission of ester linkages to oligomers leading to change in molar mass followed by further degradation to the water soluble smaller units such as monomers, dimers, and trimers with a decrease in weight. We also observed the formation of lactic acid (LA) monomer by high‐performance liquid chromatography (HPLC; Figures S4 and S5, Supporting Information). As expected, by an enhancement in degradation degree, the amounts of LA were obviously increased (**Table** **1**). The contents of LA in both kinds of water samples (SW and FW) were approximately the same.

**Table 1:** Quantification of LA in SW samples containing degraded products from PLGA by HPLC

Mass of PLGA filma) [mg]	Degradation [%]	Produced LA from films [mg]
122.10 ± 4.20	0	0
121.77 ± 6.65	48.07 ± 19.34	31.47 ± 0.90
112.77 ± 8.45	99.47 ± 0.92	61.83 ± 0.48

^a)^ Three samples were tested for each measurement.

**Figure 3:**
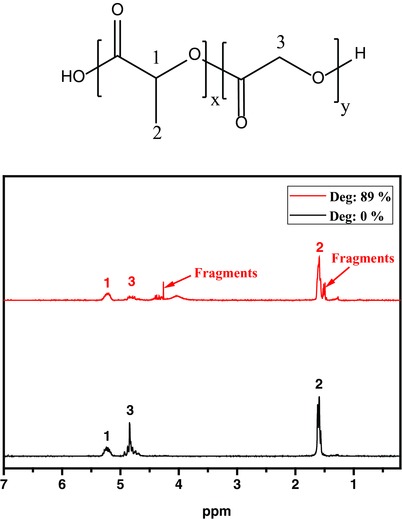
^1^H NMR spectra of PLGA before (deg: 0%) and after degradation (deg: 89%).

The effects of structural changes in PLGA polymer have been analyzed by thermal studies (thermogravimetric analysis (TGA) and differential scanning calorimetry (DSC)) during the degradation process. The thermal stability and glass transition temperature (*T*_g_) were decreased with increasing degradation degree (Figure S6, Supporting Information). The initial polymer samples showed a *T*_g_ value between 44 and 46 °C, but over time and after degradation of films in water, the *T*_g_ showed a shift to much lower values. This behavior can be interpreted that in the hydration process, the aqueous medium penetrates the polymer matrix, which results in polymer relaxation and a decrease in *T*_g_. Another interpretation is based on the decreasing molecular weight of polymer so that it can reduce the *T*_g_ of the polymer. A signal around 64–66 °C in DSC became obvious in the sample left after around 57% degradation of original PLGA (Figure S6a,b, Supporting Information). This could be due to gradually increase in the ratio of PLA block in the polymer during the degradation process, implying early degradation at poly(glycolic acid) (PGA) units. This interpretation could be clearly confirmed by the application of ^1^H NMR investigations (**Figure** **3**), where structural changes of polymer chains during degradation were investigated. The ^1^H NMR spectra of original PLGA and the material left after ≈89% degradation is shown in Figure 3. The peak integration of —CH protons of PLA and —CH_2_ protons of PGA was used for calculation of the ratio of the two units in the copolymer. The PLA:PGA ratio was 1:0.98 (molar ratio) in the original polymer. The ^1^H NMR of the sample left after ≈89% degradation showed new peaks originating from the terminal OH—CH_2_— and OH—CH(CH_3_)— formed by backbone scission and showed increased amount of PLA in comparison to PGA. This shows faster hydrolysis of more hydrophilic PGA units compared to PLA. The studies on ^1^H NMR spectra of partially degraded PLGA samples in FW and SW showed approximately the same results (data not shown here).

**Figure 4:**
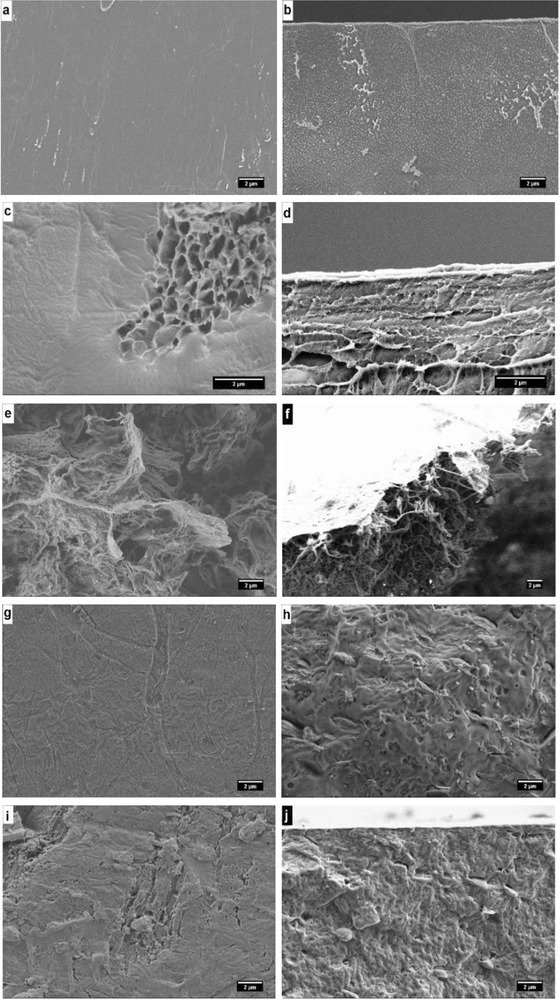
Morphological studies on the degradation of PLGA – a) surface and b) cross section of initial film, c) surface and d) cross section of the film after 16% degradation, and e) surface and f) cross section of the film after 70% degradation – and PHB – g) surface and h) cross section of initial film, and i) surface and j) cross section of the film after 8.5% degradation.

The morphologies of the PLGA films were observed before and after immersion in water for different time intervals. The original films had a smooth surface and a solid nonporous interior (**Figure** **4**a,b), which was changed with time. Both the surface and bulk showed porous structures even after ≈16% degradation on immersion in water confirming bulk degradation mechanism (Figure 4c,d). After ≈70% degradation, both the surface and bulk demonstrated clearly collapsed and perforated structures (Figure 4e,f). Additional morphological studies were also performed for PHB. PHB showed degradation by surface erosion as observed by morphological changes only on the surface (Figure 4i,j). Our morphological studies in SW and FW did not demonstrate any meaningful differences between both kinds of water sources.

## 3 Conclusion

In conclusion, a systematic study comparing degradation of different polymers under same conditions showed 100% bulk degradation of only PLGA in SW and FW in ≈270 d. PHB was degraded by ≈8% in 365 d. A significant difference between the degradation mechanism of PLGA and PHB was observed by scanning electron microscope (SEM) and GPC. PHB showed degradation by surface erosion as seen by morphological changes only on the surface and no change in molar mass in GPC after even 8.5% mass loss. The amorphous nature of the polymer might be responsible for the faster hydrolysis and complete degradation of PLGA making diffusion of water easy all throughout the bulk.

Regarding the contribution of so‐called biodegradable polymers to microplastic contamination in the environment, our results indicate that most of them do not degrade under natural conditions. However, this knowledge might be stepping stone to design novel environmentally friendly polymers for future use.

## 4 Experimental Section

Experimental section was shown in the Supporting Information.

## Conflict of Interest

The authors declare no conflict of interest.

## Supporting information

Supplementary
