# Supplementary material for: Fate of So‐Called Biodegradable Polymers in Seawater and Freshwater
Source: Glob Chall. 2017 Jun 23;1(4):1700048. doi: 10.1002/gch2.201700048 (PMC6607129; doi:10.1002/gch2.201700048)
Supplement: Supplementary file 1 — Supplementary [file GCH2-1-1700048-s001.pdf]

Copyright WILEY-VCH Verlag GmbH & Co. KGaA, 69469 Weinheim, Germany, 2017.

Supporting Information

for *Global Challenges,* DOI: 10.1002/gch2.201700048

Fate of So-Called Biodegradable Polymers in Seawater and Freshwater

Amir Reza Bagheri, Christian Laforsch, Andreas Greiner, and Seema Agarwal**

Supporting information

Fate of *so-called* biodegradable polymers in sea and fresh water

Amir Reza Bagheri, Christian Laforsch, Andreas Greiner and Seema Agarwal**

A. R. Bagheri, Prof. Dr. S. Agarwal, Prof. Dr. A. Greiner
Macromolecular Chemistry II and Bayreuth Centre for Colloid and Interfaces, University of Bayreuth, Universitätsstraße 30, 95440 Bayreuth, Germany
E-mail: agarwal@uni-bayreuth.de ; greiner@uni-bayreuth.de

Prof. Dr. C. Laforsch
Department of Animal Ecology I and BayCEER, University of Bayreuth, Universitätsstraße 30, 95440 Bayreuth, Germany

Keywords: biodegradable polyesters, freshwater, seawater, microplastics

The sources of water used in this project. In this project, we used artificial seawater (AB Reef Salt, Aqua Medic, Germany) from a coral reef aquarium (University of Bayreuth). (Figure S1.)

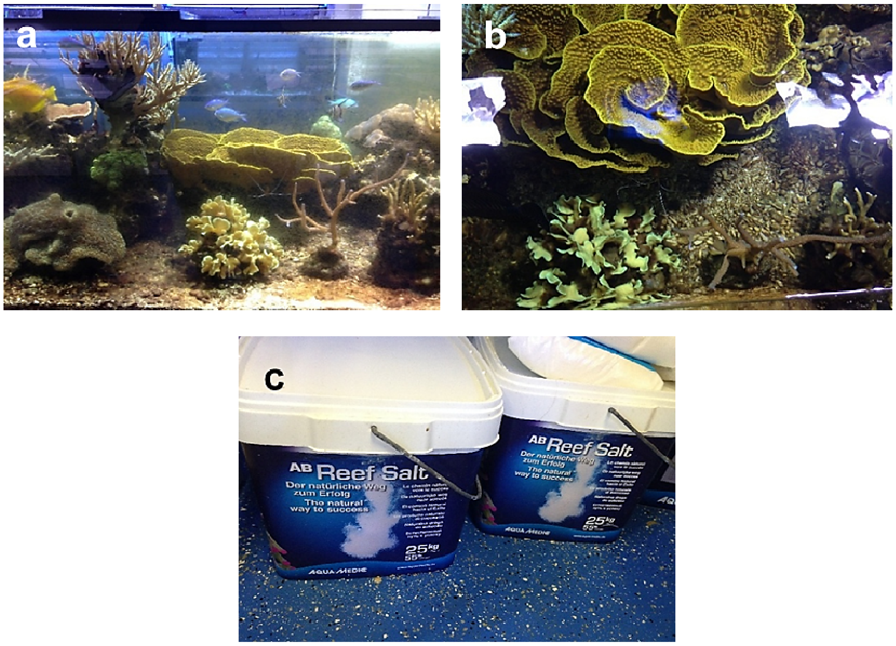

Figure S1. Artificial seawater, used in this work. a and b) artificial SW in aquariums. c) commercially available salts for the production of SW in this work.

As the freshwater, we used collected rainwater from the ponds at the University of Bayreuth (Figure S2).

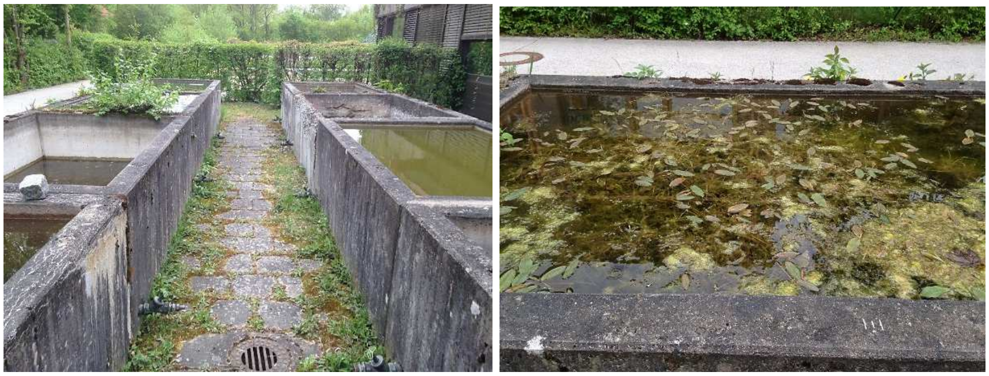

Figure S2. The freshwater from the ponds, university Bayreuth

All polymer films were immersed in 3 ml water (SW and FW) in vials which have holes on their caps so that the oxygen exchange could occur between samples and their environments. Finally, all samples were stored in the chamber at the constant temperature (25 ^{o}C) and under fluorescent light. The light was programmed so that it was switched on 16 hours per day on and 8 hours switched off. Every two weeks, all mediums were refreshed (Figure S3).

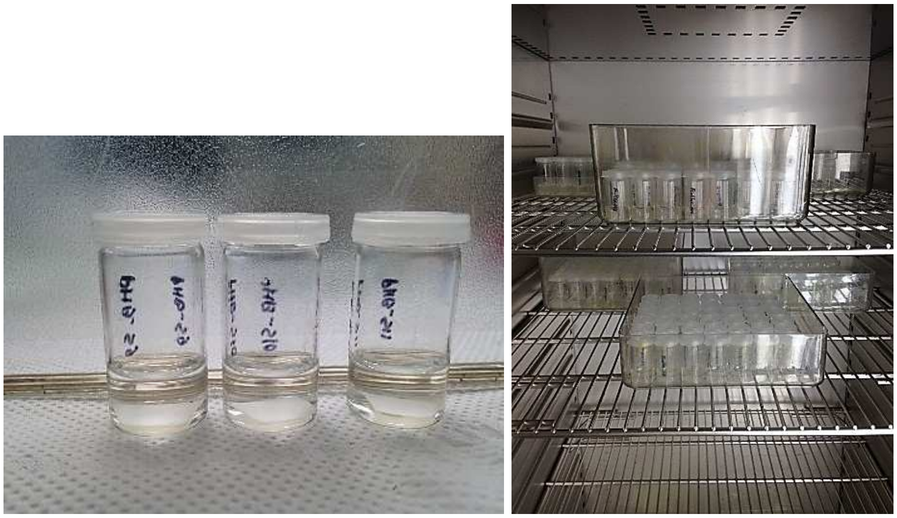

Figure S3. Left) films in capped vials with holes on the cap for air exchange, right) all samples collected in the thermostatic chamber.

The quality of seawater has been controlled every 2-3 months randomly (Table S1).

Parameters	Data range
**pH**	7.9 - 8.5
**Salinity at 25 ^{oC}**	51.0 - 54.0
**KH [^{o} dKH]**	8 - 11
**Calcium [mg/ml]**	400 - 440
**Magnesium [mg/ml]**	1200 - 1600
**Ammonium [mg/ml]**	< 0.25
**Nitrite [mg/ml]**	0
**Copper [mg/ml]**	0 - 0.3
**Nitrate [mg/ml]**	0 - 20
**Phosphate [mg/ml]**	< 0.1
**Silicate [mg/ml]**	< 1

Table S1. Quality control of seawater

Polymer	Trade name	Company	MW (gr.mol^{-1})	T_m (^oC)	T_g (^oC)
**PLGA**	Resomer RG 503	Evonik	~ 38,000	amorphous	44 to 48
**PCL**	Capa^{TM} 6800	Perstrop	~ 80,000	58 to 60	-58 to -60
**PBAT**	Ecoflex F Blend A1200	BASF	~ 100,000	110 to 120	-27 to -29
**PHB**	Polyhydroxybutyrate	Goodfellow	~ 250,000	171 to 182	5 to 10
**PLLA**	Resomer L 210 S	Evonik	~ 230,000	180 to 195	70 to 75
**PET**	PET RT32	Trevira	~ 38,000	249 to 252	74 to 77

Table S2. The specifications of polymer

The validation of HPLC method, which was used in this project for detection of LA demonstrated the linear behavior and repeatability of standard solutions of LA (Figure S4).

The concentration of released LA in water samples, after the degradation of PLGA in water, has been calculated according to standard solutions of LA.

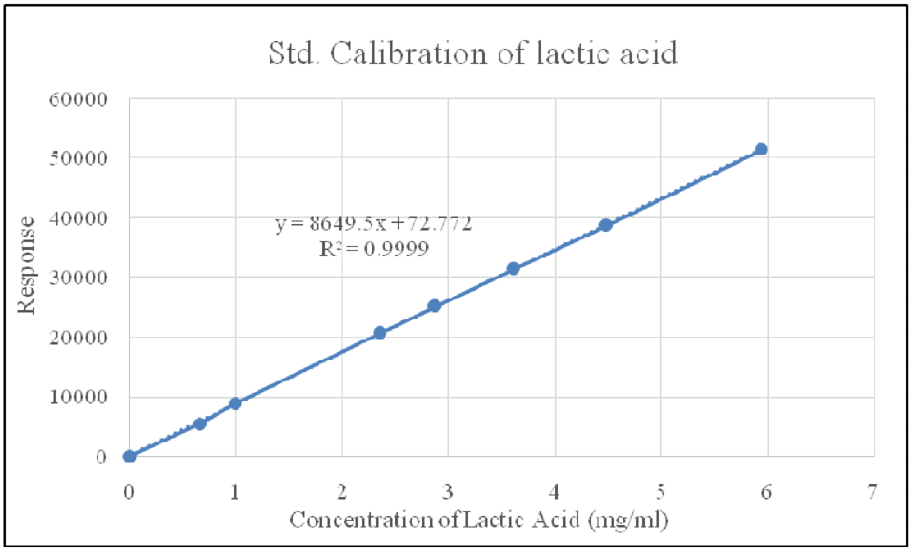

Figure S4. Validation of HPLC by calibration line of standard solutions of LA

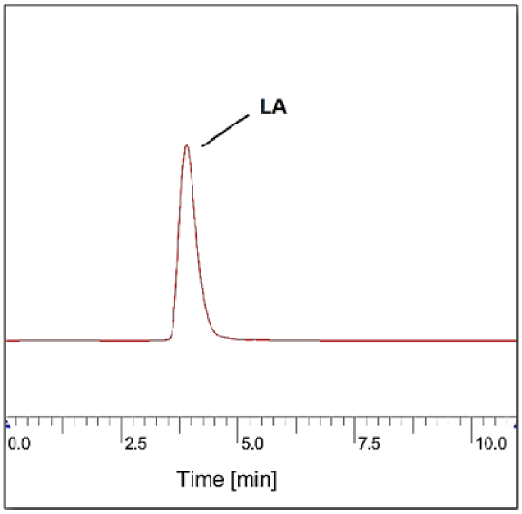

Figure S5. HPLC chromatogram of water containing LA produced from degradation of PLGA

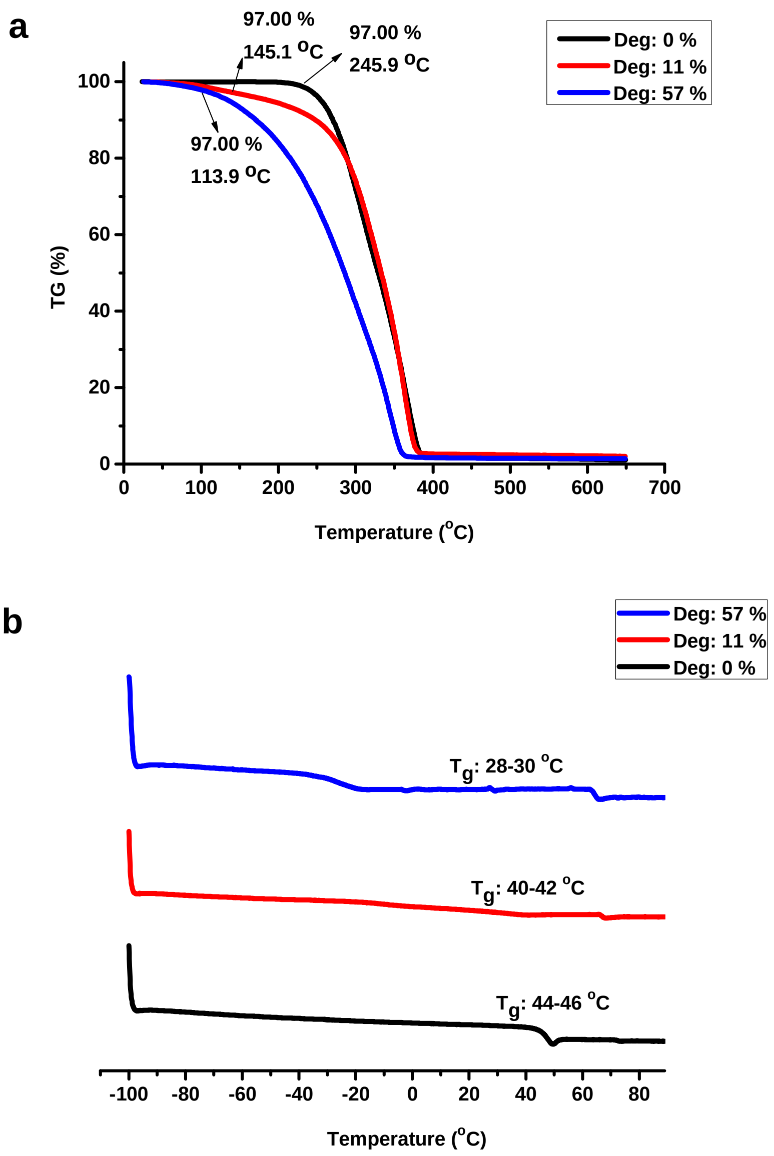

Figure S6. Thermal properties of PLGA film before and after degradation. a) TGA and b) DSC.

Instrumental characterization

TGA: Thermogravimetric analyses (TGA) were done with a TG 209 F1 (Netzsch) by heating the samples up to 650 °C, under a nitrogen atmosphere. The heating rates of 10 K min^{-1} and about 5–10 mg of each sample was applied.

DSC: Differential scanning calorimetry (DSC) measurements were performed by a Mettler thermal DSC analyzer (821c) used for the thermal characterization of PLGA. The DSC experiments were accomplished in a nitrogen atmosphere and heating rates of 5 K min^{-1}. The temperatures were adjusted between -100 to 100 °C.

NMR: Nuclear magnetic resonance (NMR) spectroscopy was conducted in $CDCl_3$ as a solvent using a Bruker ARX300 spectrometer and MestReNova software for evaluation.

GPC: Gel permeation chromatography (GPC) was carried out in chloroform as the eluent at a flow rate of 0.5mL.min^{-1} at room temperature, a pre-column PSS SDV (particle size 5 μm) and a column PSS SDV XL linear (particle size 5 μm) calibrated against polystyrene standards (PSS) using a PSS SECcurity RI detector. The GPC data were analyzed by the software PSS WinGPC Unity, Build 1321.

HPLC: High-performance liquid chromatography was performed using Waters system equipped with autosampler AS100 and waters 2489 as UV detector.

SEM Microscopy: Scanning electron microscope (SEM) was conducted using the electron microscope model LEO 1530. ImageJ software was applied for evaluation of micrographs.

Materials

PLGA (Resomer RG 503) was prepared from Evonik co., PCL (Capa^{TM} 6800) from Perstorp Co., Ecoflex (Ecoflex F Blend A1200) from BASF Co., PLA (Resomer L 210 S) from Evonik Co. and PET (PET RT32) from Trevira Co. PHB was purchased from Goodfellow Cambridge Ltd Co. Acetonitrile was purchased from VWR Co. and phosphoric acid from Grussing GmbH. All other solvents and chemicals were prepared from Sigma-Aldrich Co.

Experimental Section

Fabrication of polymer films: All polymeric films were produced by heat pressing process. All these previously mentioned polymers were heated up to approximately their melting points. Before heating, the required quantities of each polymer were put in a template with a thickness of 300 μm then heated up and pressed.

Controlled degradation studies: All films (average dimension: 1.2 x 1.2 cm and average thickness: 320 ± 20 μm) were placed in snap cap vials. All vials were filled then either by three ml freshwater or seawater. All polymers in water were stored in the thermostatic chamber at the constant temperature of 25 ^{o}C and under the white lamp. Mediums were refreshed by three ml new mediums every two weeks. The contaminated mediums were collected, centrifuged and filtered. All filtered water samples were finally stored in the cooling room (2-5 ^{o}C) to investigate the possible quality tests. At desired time periods, some films of each polymer were taken out and washed several times with distilled water and then frozen in liquid nitrogen and dried by freeze-dryer. By comparison between the weights before and after degradation, the mass loss (%) for each film could be earned. For each period, three samples of each polymer have been taken out, and end-results have been reported as the average ± standard deviation (SD).

Determination of lactic acid (LA) in water: The contents of LA in water samples could be determined by HPLC. The separation was done on the reversed phase C18 column, Eclipse XDB-C18 150 mm×4.6 mm with particle size 5 μm using a mobile phase mixture buffer (phosphoric acid 0.02 M in water) and acetonitrile in a ratio of 88:12 v/v and the elution was isocratic at a flow rate of 0.4 mL/min. The determinations were performed with a UV-Vis detector at 200 nm. The volume of the injected sample was 20 μL. The temperature of the column was adjusted at 35 ^{o}C. The amounts of released LA have calculated accumulatively for three films after predetermined time periods.
